# Maximizing Kolmogorov Complexity for accurate and robust bright field cell segmentation

**DOI:** 10.1186/1471-2105-15-32

**Published:** 2014-01-30

**Authors:** Hamid Mohamadlou, Joseph C Shope, Nicholas S Flann

**Affiliations:** 1 Department of Computer Science, Utah State University, Logan, UT 84322, USA; 2 Institute for Systems Biology, Seattle, WA 98109, USA; 3 Synthetic Biomanufacturing Institute, Logan, UT 84322, USA; 4 Department of Biology, Utah State University, Logan, UT 84322, USA

## Abstract

**Background:**

Analysis of cellular processes with microscopic bright field defocused imaging has the advantage of low phototoxicity and minimal sample preparation. However bright field images lack the contrast and nuclei reporting available with florescent approaches and therefore present a challenge to methods that segment and track the live cells. Moreover, such methods must be robust to systemic and random noise, variability in experimental configuration, and the multiple unknowns in the biological system under study.

**Results:**

A new method called *maximal-information* is introduced that applies a non-parametric information theoretic approach to segment bright field defocused images. The method utilizes a combinatorial optimization strategy to select specific defocused images from each image stack such that set complexity, a Kolmogorov complexity measure, is maximized. Differences among these selected images are then applied to initialize and guide a level set based segmentation algorithm. The performance of the method is compared with a recent approach that uses a fixed defocused image selection strategy over an image data set of embryonic kidney cells (HEK 293T) from multiple experiments. Results demonstrate that the adaptive *maximal-information* approach significantly improves precision and recall of segmentation over the diversity of data sets.

**Conclusions:**

Integrating combinatorial optimization with non-parametric Kolmogorov complexity has been shown to be effective in extracting information from microscopic bright field defocused images. The approach is application independent and has the potential to be effective in processing a diversity of noisy and redundant high throughput biological data.

## Background

Cell segmentation is the identification of cell objects and their observable properties from biological images. Current cell segmentation methods perform most accurately when applied to high contrast and minimal noise images obtained from samples where the cells have fluorescently-labeled cell nuclei and stained membranes, and are distinct with minimal adherent membranes. However, these ideal conditions rarely exist.

Fluorescently tagging cells using green fluorescent protein (GFP) leads to robust identification of each cell during segmentation. While GFP tagging is widespread, there are disadvantages when applying the method repeatedly to the same sample since under repeated application of high-energy light the cells can suffer phototoxicity. Such light can disrupt the cell behavior through stress, shorten life and potentially confound the experimental results [1-3]. Significantly, a requirement for GFP labeling adds a step before a new cell line can be studied, thus making it difficult to apply this method in a clinical setting.

The alternative is to use bright field microscopy, the original and the simplest microscopy technique, wherein cells are illuminated with white light from below. However, using only bright field imaging of unstained cells presents a challenging cell detection problem because of lack of contrast and difficulty in locating both cell centers and borders, particularly when cells are tightly packed. Bright field imaging, while eliminating phototoxicity, leads to an excess of segmentation errors that significantly reduce biological and medical utility.

We seek to remedy the disadvantages and harness the experimental advantages of bright field microscopy of living cells by applying information-theoretic measures over defocused images to improve segmentation accuracy. The approach applies Kolmogorov complexity to identify the most informative subset of images within the focal stack that maximize information content while minimizing the effect of noise.

The paper first briefly reviews existing methods for segmentation of living cells, with a focus on recent approaches to defocused bright field images. Next, measures of Kolmogorov complexity are introduced and applied to image data. The new *maximal-information* method is then defined and evaluated by comparing its performance with a recent method *sephaCe*[3] over image sequence data sets from three separate experiments. An analysis and a discussion of the results follows.

### Cell segmentation methods

Several cell segmentation approaches have been developed over time for detection of live cells in microscopy images [4-7]. Most of the approaches binarize an image with certain thresholding techniques, and then use a watershed or level-set based method on either intensity, gradient, shape, differences in individual defocused images (referred to as frames) [3,8], or other measures. The algorithms then remove small artifacts with size filters, and apply merge and split operations to refine the segmentation [4-6].

#### Florescent microscopy cell segmentation

Most studies can primarily be categorized into a few key approaches. Wavelets are used for decomposing an image in both the frequency and spatial domain, and can be an effective tool since wavelets are robust to local noise and can discard low frequency objects in the background. Genovesio et al. [9] developed an algorithm to segment cells by combining coefficients at different decomposition levels. Wavelet approaches work well with whole cell segmentation, but have difficulty to segment internal cell structures. In Xiaobo et al. [10] a watershed algorithm was introduced for cell nuclei segmentation and phase identification. Using adaptive thresholding and feature extraction, Harder et al. [11] classified cells into four cell classes comprising of interphase cells, mitotic cells, apoptotic cells, and cells with clustered nuclei. In Solorzano et al. [12] the level set method determines cell boundaries by expanding an active contour around each detected cell nuclei.

While these cell segmentation algorithms have been developed for fluorescence microscopy images, defocused bright field cell segmentation demands more complex and advanced level of image processing. Broken boundaries, poor contrast, partial halos, and overlapping cells are some of the shortcomings of available algorithms [3,8] when applied to images lacking fluorescent reporters.

#### Defocused bright field microscopy approaches

Selinummi et al. [13] introduced z-projection based method to replace whole cell florescent microscopy with bright field microscopy. This method computes an intensity variation over a stack of defocused images (referred to as the z-stack) to obtain a contrast-enhanced image called a z-projection. Since variability of pixel intensity inside a cell is high compared to the background, the resulting z-projection image has high contrast and can substitute for an image obtained through whole cell florescent microscopy. The z-projection approach is straightforward and free from parameters setting. However, in order to distinguish between adherent cells, a second channel of nuclei florescent microscopy is required. As a final step *CellProfiler*[14] software is applied to both the z-projection and nuclei florescent channel to produce cell segmentation. While the z-projection approach avoids whole cell florescence, it still requires an additional nuclei channel of florescent microscopy and so does not eliminate potential problems with cell toxicity.

## Implementation

A recent method that needs only bright-field defocused images has been introduced in *sephaCe*[3]. This system is capable of both the detection and segmentation of adherent cells and can be downloaded from (http://www.stanford.edu/~rsali/sephace/seg.htm) as a free and open source image analysis package. In contrast to Selinummi et al. where all the frames of the z-stack are utilized, *sephaCe* selects only a subset of five frames as input to the image processing system. *sephaCe* selects this subset using a hard-coded strategy independent of each data set and each individual z-stack contained within that data set. Therefore *sephaCe* does not adapt to the inevitable equipment and biological sample variation. While parameters of the image processing method can be tuned for specific data sets somewhat ameliorating the problem, a more general purpose non-parametric frame selection method is needed for high-throughput processing of diverse data sets. This work introduces a new adaptable frame selection method that applies an information theoretic measure to select frame subsets specific to the idiosyncracies of each z-stack. This method is referred to as *maximal-information*.

Following frame subset selection, the *maximal information* method applies the same image processing and segmentation algorithm of *sephaCe*. Ali et al. [3,8] presents a series of algorithms that automatically segment each z- stack without the need for any florescent channel. The key to discriminating adherent cells is to initialize a level-set algorithm [15] with the difference between two strongly defocused frames and then guide contour expansion using the difference of two weakly defocused frames. As an initial step, the in-focused frame is detected by selecting that image from the z-stack in which the Shannon entropy [16] is minimized. Given an image histogram *I*, entropy is defined as:

(1) $$E(I)=-\int_{y=1}^{n}\int_{x=1}^{m}p(I(x,y))\;\text{log}\;p(I(x,y)))\text{dxdy}$$

Where *p*(*I*(*x*,*y*)) is the probability of pixel intensity values. Entropy value is expected to be maximized for strongly out of focused images and minimized for the in-focus image. Let the in-focus image frame be *I*^0^.

After detecting the in-focus image, four additional images from the z-stack are selected, two above the in-focus frame and two below. To initialize the level set algorithm, a difference image is generated from two strongly defocused images selected at a fixed distance of ±25 *μm* from the in-focus frame, referred to as *I*^++^ and *I*^--^. This image is binarized using the Otsu [17] thresholding method and then small artifacts are removed by labeling connected components and applying size filter.

To guide the level set algorithm in expanding the initial cell boundaries, another difference image is generated between two slightly defocused images ±10 *μm* from the in-focus frame, referred to as *I*^+^ and *I*^-^. Details on how this difference image is applied to compute local phase and local orientation images that direct the border expansion is given in [8] and [3].

### Motivation for the maximal information approach

**Figure 1:**
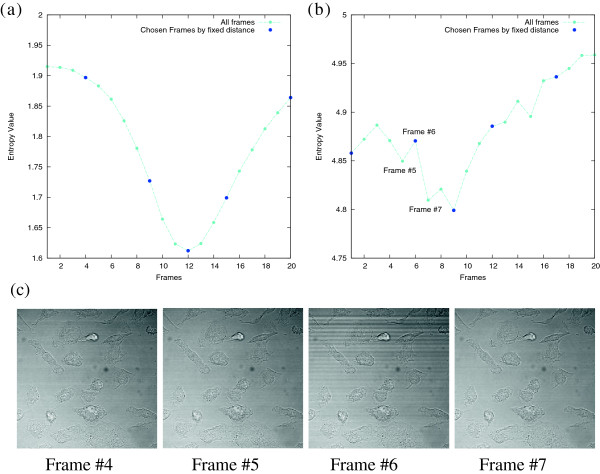
**Relationship between frame entropy as the focus level changes in the z-stack is shown in (a) and (b).** In **(a)** there is a monotonic increasing and then decreasing relationship between focus and entropy, with the in-focus frame containing minimum entropy. In **(b)** a nosier data set is employed and the relationship between focus and entropy is irregular. As can be seen in frame 6, banding and stripe noise introduced by the microscope unexpectedly increases entropy. **(c)** Illustrates four corresponding frames for data set analyzed in graph (b).

In the *sephaCe* package the four defocused frames are chosen at fixed distances (±10 *μm*,±25 *μm*) from the in-focused frame to initialize and guide the level-set algorithm. Figure 1(a) illustrates an entropy analysis of a z-stack with 21 frames in which the image separation is 3 *μm*. The in focus frame *I*^0^ is determined as the 12’th frame, the 9’th and 15’th frames are the weakly defocused frames *I*^-^ and *I*^+^ (in this case ±9 *μm* due to sampling resolution), the strongly defocused frames *I*^--^ and *I*^++^ are the 4’th and 20’th frames. In this z-stack image, as the frames become more blurred, their entropy increases monotonically implying that there are no irregularities within the frames. In this ideal case, the fixed strategy can produce reasonable results.

However, in experiments over a diversity of images (given in Section Results) this fixed selection of out-of-focus frames is demonstrated to produce poor segmentation. A fixed strategy cannot take into account random and systemic noise, variability in experimental configurations including microscope configurations, and multiple unknowns in the biological system under study. Some of these conditions are illustrated in selected frame images in Figure 1(c). Two possible reasons to account for the irregular entropy-focus plane relationship in Figure 1(b) are: 

• Biological variability where cells do not adhere to the flat surface of the culture medium but vary in the z-dimension as they change morphology and form cell-cell adhesive bonds. That is, a focused frame for one cell could be a defocused frame for other cells. In Figure 1(c), the bright upper cell is positioned higher than the rest. Therefore a semi-random level of sharpness resides in the all defocused images.

• Systemic noise introduced by microscopy and imaging. For instance in Figure 1(c), frame 6 has strip noises introduced by the camera. Strip noise residing in the image increases the entropy value from the 5’th frame to 6’th frame while a decrease is expected.

Applying this fixed distance strategy to select strongly defocused frames can add unwanted initial active contours resulting in over-segmentation and also can miss initial active contours resulting in under-segmentation. Likewise, fixed selection of weakly defocused frames can add anomalies into the local phase and orientation images and thus misdirect the contour expansion to include or exclude cells, particularly when cells are tightly packed.

Overall, the fixed approach in selecting initial images in the *sephaCe* package is brittle and error-prone. The unavoidable variation requires an *adaptable* method rather than a fixed approach. The *maximal-information* method uses an optimization based approach that searches the combinations of z-stack frames to select the four frames that contain the highest information, evaluated using Kolmogorov information-theoretic measure [18]. This process is repeated for each individual z-stack and so adapts to the distinctiveness of each sample. Since the *maximal-information* method is adaptive, it can be applied to a diversity of data sets utilizing different microscopes, lighting conditions and biological samples.

### Kolmogorov information set complexity

Set complexity [19], denoted *Ψ*, is applied to quantify the amount of information contained within each possible set of four image frames. The measure is general purpose and non-parametric in that it computes the information content of set of objects so long as they can be encoded as strings. Set complexity has been applied to understand the organization and information content of biological data sets including developmental pattern formation [20], genetic regulatory network dynamics [21], and gene interaction network structure [22]. The Kolmogorov complexity [18] of a string is the length of shortest algorithm that can be used to generate the string. Exact computation is undecidable, but it can be approximated by the compression size of a string. Bzip2 and zip compressor with block size of 900 Kbytes have been tested and shown robust for this purpose.

A related Kolmogorov complexity measure is the Normalized Compression Distance *NCD*) defined as the length of the shortest program that computes one given string from another. This measure provides a quantification of similarity between the strings since the more similar they are, the shorter the program needed. Again, this measure is undecidable but can be estimated using compression. Normalized Compression Distance described in [23] and [24] defined below, is such a measure of similarity between two objects that applies compression size *C*(*s*) of string *s*:

(2) $$\begin{array}{c} \text{NCD}(s_{i},s_{j})=\frac{C(s_{i}+s_{j})-\min(C(s_{i}),C(s_{j}))}{\max(C(s_{i}),C(s_{j}))} \end{array}$$

Where *s*_*i*_+*s*_*j*_ is the concatenation of *s*_*i*_ and *s*_*j*_ string. If the two strings compress smaller together than separately, then *NCD* will be closer to 0.0. As the two strings are more similar, the concatenated string is more compressed resulting in a lower *NCD* value. Random strings or dissimilar regular patterns are not as compressed and so *NCD* will be closer to 1 [25,26]. 

1. $C(s_{i}^{s}+s_{j}^{s})≃C(s_{i}^{s})≃C(s_{j}^{s})$ then $\text{NCD}(s_{i}^{s},s_{j}^{s})≃0.0$

2. $C(s_{i}^{r}+s_{j}^{r})≃C(s_{i}^{r})+C(s_{j}^{r})$ then $\text{NCD}(s_{i}^{r},s_{j}^{r})≃1.0$

3. $C(s_{i}^{r}+s_{j}^{s})≃C(s_{i}^{r})$ and $C(s_{j}^{s})≃0.0$ then $\text{NCD}(s_{i}^{r},s_{j}^{s})≃1.0$

Where *s*^*r*^ is from the set of random strings and *s*^*s*^ are simple strings containing regular patterns.

Set complexity [19] of a set of *n* strings *S*={*s*_1_,…,*s*_*n*_} is defined:

(3) $$\;\Psi (S)=\frac{1}{n(n-1)}\underset{s_{i}\in S}{\sum }C(s_{i})\underset{s_{j}\neq s_{i}}{\sum }\text{NCD}(s_{i},s_{j})(1-\text{NCD}(s_{i},s_{j}))$$

Set complexity captures the relationships among strings in the set, discounting when strings are very similar (*NCD* close to 0.0) and so contain the same information, or highly dissimilar so that they have nothing in common and appear random (*NCD* closer to 1.0). The value is maximized when each string is intrinsically complex (high *C*(*S*_*i*_)) and the similarity between the strings lies between maximally dissimilar and maximally similar *NCD*(*s*_*i*_,*s*_*j*_)≃0.5, which occurs when *C*(*s*_*i*_+*s*_*j*_)≃*C*(*s*_*i*_)/2-*C*(*s*_*j*_), assuming *C*(*s*_*i*_)>*C*(*s*_*j*_).

**Figure 2:**
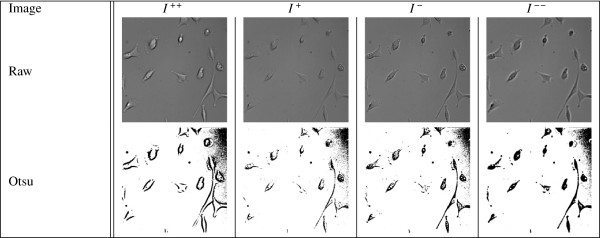
**Strongly and weakly defocused selected frames from time step 1 in data set one.** Top row is the raw image frames. The second row is the binary image following Otsu thresholding that is linearized and compressed.

Figure 2 gives an example of applying *Ψ*(*S*) to defocused images. Along the top are the original frames and below them is their binary representation following an Otsu thresholding step. Each binary image is encoded as a string by concatenating each column scanning from left to right (more details are provided in Algorithm 1). For each image the compression size is given. *NCD* values between each pair of the images is provided in Table 1.

**Table 1:** **The *NCD *** **values for the four image frames given in Figure **2

**NCD**	***I***^**++**^	***I***^**+**^	***I***^**-**^	***I***^**--**^
*I*^++^	0.0	0.1429	0.2154	0.1071
*I*^+^	0.0	0.0	0.2615	0.1296
*I*^-^	0.0	0.0	0.0	0.2000
*I*^--^	0.0	0.0	0.0	0.0

### The maximal-information segmentation method

To select the four most informative frames from a z-stack with *n* frames, the method searches the space of all possible combinations of two frames from above the in-focus frame (*I*^++^ and *I*^+^) and two frames from below the in-focus frame (*I*^-^ and *I*^--^), evaluates each set for *Ψ*, then picks the maximizing combination. The method is given in Algorithm 1.

#### Algorithm 1 **The *maximal-information *** **algorithm to select the four z-stack frames needed to initialize the level set method for segmentation. Let the input z-stack be *I *** **containing *n *** **frames. The algorithm returns the in-focus frame and four defocused frames. Note that all compression calculations are calculated once and cached.**

First each image in the z-stack is binarized using the Otsu [17] thresholding method and then converted to a string (linearization) by concatenating each column of the image to the next column [27]. Many methods of linearization were explored in [27] and column concatenation was found to be effective because spatially located regularities are picked up by compression. Bzip2 is applied to compute the compression size of each individual string and also each pairwise concatenated string (for *NCD*, Equation 2). From these cached compression values, pairwise *NCD* values are determined.

The *O*(*n*^2^) compression step dominates the computation time since strings must be written to file before processing; the final *Ψ* calculation involves only matrix operations and is very fast, even though more combinations must be computed. For the three data sets studied in this work, the preprocessing and level set algorithms of *sephaCe* take approximately 10 seconds per z-stack. The *maximal-information* frame selection method adds approximately 20 seconds per z-stack to the run time. Timings were on an Intel Pentium G640 Processor 2.8 GHz (3 MB cache).

## Results

### Set complexity analysis of image data

To understand how Kolmogorov Complexity measures could reveal information in z-stacks, an initial study was performed by computing the *NCD* between each pair of 21 frames for three data sets each containing 192 z-stacks. The data sets used for in this work are human embryonic kidney cells (HEK 293T) sampled at 5 minute intervals for 16 hours. Each z-stack sequence is from a distinct experiment. Data was obtained using a Leica DM6000 microscope with each z-stack containing 21 image frames each separated by 10 *μm*, with resolution 1024 × 1024 12-bit grey-scale pixels. Since the z-stack was sampled at a 10 *μm* resolution, the strongly defocused frames for *sephaCe* were set at ±30 *μm*.

**Figure 3:**
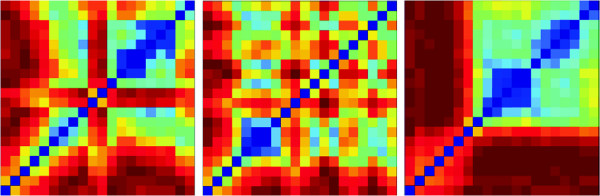
***NCD *values shown as a heatmap for all pairs of image frames in the z-stack of three selected defocused image stacks from the same experiment.** Color code blue specifies pairs of frames with lowest *NCD* values and red specifies highest *NCD* values. In each heatmap, the lowest z frame is in the lower left, the highest z frame is in the upper right. Analysis illustrates that off-diagonal *NCD* values range from 0.6 (most similar images) to 1 (red, most dissimilar images). Along the diagonal *NCD* equals zero (blue). Note the diversity of similarity relationships among the frames of each z-stack.

Figure 3 presents values of *NCD* in the form of a heatmap for each pair of frames along the z-stack sequence for a selection of three images. Frames tend to decrease in similarity as the focus distance increases so that blue areas (low *NCD*) are mostly around the diagonal, and red areas off the diagonal. However, each image displays significant individuality due to noise, microscope variability over time and changes in the biological sample as cells divide, die and move. This inconsistency among *NCD* matrices over time justifies the need for an adaptive frame selection strategy.

**Figure 4:**
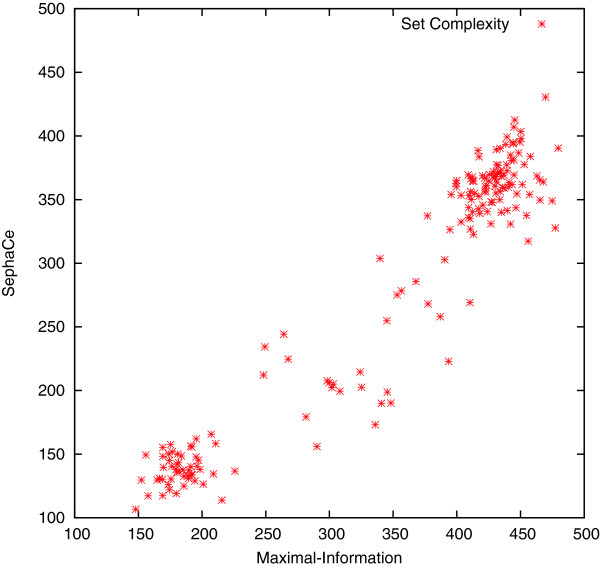
**A parametric plot of set complexity values for the four defocused frames selected by the two algorithms.** The *X* axis indicates the complexity value of the frame set selected by *maximal-information* and the *Y* axis indicates complexity value for the frame set selected by *sephaCe*. Each data point represents one z-stack from the 192 z-stacks in the human embryonic kidney cells (HEK 293T) data set.

Four frames of the z-stack are chosen to start and guide the level set algorithm. Figure 4 compares the computed *Ψ* of frames obtained by the *maximal-information* method with the *Ψ* of the frames identified using the fixed distance method of *sephaCe*, for all 192 z-stacks. In all cases the *maximal-information* frame set has a higher information content then the fixed *sephaCe* set. While this result is not surprising, it supports the need for adaptability as it demonstrates the inability of a fixed strategy to pick those images that have high intrinsic information. A mean difference hypothesis statistical analysis demonstrates that these differences are significant, see Table 2. According to the p-value in Table 2, that is much lower than 0.05, the mean difference hypothesis is rejected and so there is a significant difference between the mean values of the two groups. That is, selecting images using *maximal-information* guarantees sets with higher *Ψ* than the *sephaCe* method.

**Table 2:** Set complexity values for two different approaches

	**Fixed defocused distance (*sephaCe*)**	**Selected by *maximal-information***
Mean	278.5049	345.1289
Variance	10620.73	12336.47
Observations	192	192
Pearson correlation	0.9603	
P(T<=t) one-tail	1.19825E-67	
t Critical one-tail	1.6536	
P(T<=t) two-tail	2.3965E-67	
t Critical two-tail	1.9736	

### Precision and recall analysis

**Figure 5:**
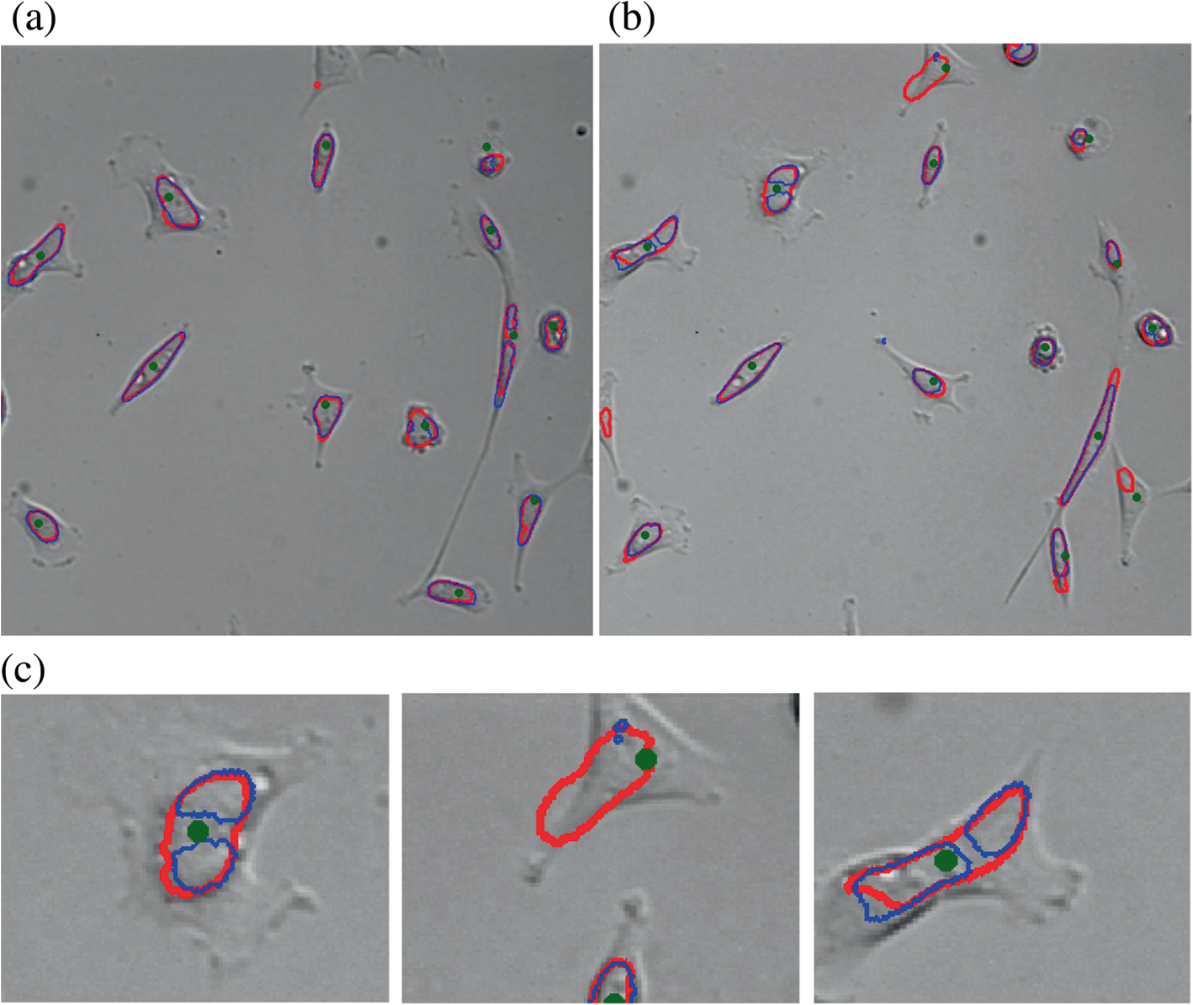
**Example cell segmentation results for two z-stacks of human embryonic kidney cells (HEK 293T) overlaid on the in-focus frame.** Segmentations produced by *maximal-information* are shown in red; segmentations produced by *sephaCe* are shown in blue. In **(a)** both algorithms select similar frames and produce similar and accurate results. In **(b)***maximal-information* selects a alternative set of frames at different focus planes from the fixed strategy and produces significantly lower segmentation errors. Here the *sephaCe* method fails to accurately detect four cells along with over segmenting another. In **(c)** segmentation results are shown closeup.

Two examples of segmented bright field microscopy frames are shown in Figure 5. In (a) both algorithms select similar frames and produce similar and accurate results. In (b) *maximal-information* selects a alternative set of frames at different focus planes (compared to the fixed strategy) and produces significantly lower segmentation errors. Here the *sephaCe* method fails to accurately detect four cells along with over-segmenting another.

In order to evaluate the segmentation results, the raw microscope z-stacks were provided to a human expert (Joseph C. Shope, Utah State University) who identified the cells using *Image-Pro Plus* (Media Cybernetics). Optimal z-frames were selected and cell centers determined by fitting a major and minor axis to produced excel files of cell center coordinates for each z-stack. No segmentation results were given to the expert during this initial cell identification. In parallel, the two methods were applied to the data sets to produce segmentation results for each z-stack, drawn as overlays with red (*maximal-information*) and blue (*sephaCe*) as in Figure 5. Next, the segmentation results were overlaid with the expert-determined cell centers and for both methods a count was made of the correctly identified cells (true positive), missing (false negative) and fragments of cells identified as one cell or spurious objects (false positive). To measure the quality and utility of the methods overall, the precision *Pr* and recall *Re* of *maximal-information* and *sephaCe* correction was determined, where:

$$\text{Pr}=\frac{\text{tp}}{\text{tp}+\text{fp}}\;\;\;\;\text{Re}=\frac{\text{tp}}{\text{tp}+\text{fn}}$$

with *tp*, *fp*, *fn* being the count of detected true positive, false positive, and false negative objects, respectively. In Table 3 the precision and recall of *maximal-information* are both significantly better than *sephaCe* for each of the three data sets.

**Table 3:** Segmentation results for three data sets for human embryonic kidney cells (HEK 293T)

**Data set one**	***Maximal-information***	***SephaCe***	***Correlation***	**t- stat**	***P*** **(*T *** **≤ *t*)one-tail**
Correct segmentation *tp*	9.12	5.76	0.3970	9.4557	0.0
Unexpected areas *fp*	0.68	0.80	0.2355	-0.5492	0.2939
Missing cells *fn*	1.60	4.72	-0.0909	-9.0929	0.0
Precision *Pr*	93.20%	89.36%	0.3295	1.4461	0.0805
Recall *Re*	85.37%	54.34%	-0.2903	8.2830	0.0
**Data set Two**	***Maximal-information***	***SephaCe***	**Correlation**	**t stat**	***P*** **(*T *** **≤ *t*)one-tail**
Correct segmentation *tp*	13.35	12.60	0.4344	3.4701	0.0012
Unexpected areas *fp*	1.15	2.20	0.1633	-4.0977	0.0003
Missing cells *fn*	0.50	1.25	0.2939	-3.4701	0.0012
Precision *Pr*	92.30%	85.45%	0.1690	4.3714	0.0001
Recall *Re*	96.40 %	91.08%	0.2822	3.4407	0.0013
**Data set three**	***Maximal-information***	***SephaCe***	**Correlation**	**t stat**	***P*** **(*T *** **≤ *t*)one-tail**
Correct segmentation *tp*	15.56	11.86	0.4549	10.18	0.0
Unexpected areas *fp*	1.72	2.00	0.3642	-0.9434	0.1759
Missing cells *fn*	2.81	6.36	0.4926	-9.9501	0.0
Precision *Pr*	91.66%	86.23%	0.3887	2.6898	0.0
Recall *Re*	85.94%	65.21%	0.4256	10.12	0.0

*tp* is the average count of correctly identified cells, *fp* is unexpected segmentations and *fn* is cells that were missed. Recall and precision are given as percentages.

In Table 3 the average correctly segmented cells for *maximal-Information* is higher than *sephaCe* method and demonstrates the advantage of extracting more informative frames in the z-stack. The average of both missing and unexpected cell segmentation for *maximal-information* are lower than *sephaCe* method. All three of these measures of quality are shown to be significantly better for *maximal-information* than for the *sephaCe* using a paired one-tail T-test (values that are less than 10^-8^ are reported as 0.0 in the table).

In addition, Table 3 includes the inter-method correlation of *tp*, *fp*, *fn* over the z-stack data sets. High correlation implies that the performance of both methods is consistent in that they perform poorly on the same set of “difficult” images, and well on the same set of “easy” images. Results in Table 3 show that true positives are highly correlated implying that the cells correctly identified by *maximal-information* include some of the set of cells recognized by *sephaCe*.

## Conclusions

This work has presented a method for identifying live cells in bright field defocused images. The method applies Kolmogorov complexity measures to identify specific out-of-focus frames that encode the maximum information. These frames are then used to initialize active contours and guide contour expansion for level-set segmentation algorithms as applied in the *sephaCe* method.

The new *maximal-information* approach is compared with a selection strategy employed in the original *sephaCe* that picks out-of-focus frames using fixed offsets from the estimated in-focus frame. An empirical study using a large data set of embryonic kidney cells (HEK 293T) z-stacks taken from different experimental runs has demonstrated that the adaptive method significantly improves the recall and precision of the segmentation.

Kolmogorov set complexity identifies the most informative frames by exploiting similarity measures between all pairs of frames contained within the *NCD* matrix. Each selected frame is sufficiently dissimilar (high *NCD*) to other frames in the set so as to provide unique and synergistic information about each cell in the z-stack. Recall that the dissimilarity is due to changes in cell appearance as the focal plane is moved through the cell profile. By selecting the best degree of dissimilarity, the differences between frames (used to initialize and guide the active contour of the level-set method) maximize sensitivity to the presence and shape of cells. Kolmogorov set complexity also tempers the effects of noise by discounting frames that have too higher dissimilarity since this is most likely due to noise.

The method introduced here is generally applicable because it relies on fundamental non-parametric information-theoretic properties and treats data as simple strings, ignoring the actual semantics. Robustness is achieved by viewing frame selection as combinatorial optimization problem with set complexity as the scoring function. The full potential of the method in dealing with noise, variability in experimental configurations, and multiple unknowns across a diversity of biological data will be explored in further studies.

## Availability and requirements

**Project name:** maximal-information

**Project home page:**https://sites.google.com/site/maximalinformation,

**Operating system(s):** Platform independent

**Programming language:** Matlab

**Other requirements:** requires *sephaCe*[3] downloadedfrom (http://www.stanford.edu/~rsali/sephace/seg.htm

**License:** GNU GPL

**Any restrictions to use by non-academics:** Contactcorresponding author

## Competing interests

The authors declare that they have no competing interests.

## Authors’ contributions

HM and NSF conceived the method and wrote the manuscript. HM wrote the code and performed all the computational experiments. JS assisted with the writing, analyzed all the raw images and evaluated performance. All the authors have read the paper and approve its contents.
